# Predicting the efficacy of chemoradiotherapy in advanced nasopharyngeal carcinoma patients: an MRI radiomics and machine learning approach

**DOI:** 10.3389/fonc.2025.1554899

**Published:** 2025-06-26

**Authors:** Liucheng Chen, Zhiyuan Wang, Ji Zhang, Ying Meng, Xuelian Wang, Cancan Zhao, Longshan Shen

**Affiliations:** ^1^ Department of Radiology, The First Affiliated Hospital, Bengbu Medical University, Bengbu, Anhui, China; ^2^ Department of Radiology, The Second Affiliated Hospital, Bengbu Medical University, Bengbu, Anhui, China

**Keywords:** nasopharyngeal carcinoma, radiomics, machine learning, efficacy prediction, magnetic resonance imaging

## Abstract

**Background:**

Machine learning methods play an important role in predicting the efficacy of chemoradiotherapy in patients with nasopharyngeal carcinoma (NPC). This study explored the predictive value of machine learning models based on multimodal magnetic resonance imaging (MRI) radiomic features for the efficacy in patients with advanced NPC after clinical chemoradiotherapy.

**Methods:**

A retrospective analysis was conducted on data from 160 diagnosed patients with NPC confirmed by pathology at the First Affiliated Hospital of Bengbu Medical College. Patients were divided into effective group (n=116) and noneffective group (n=44) according to the Response Evaluation Criteria in Solid Tumors 1.1 (RECIST 1.1). After the overall Synthetic Minority Over-sampling Technique (SMOTE) sample balance, the proportion of effective group and invalid group is 1:1, both 116 cases, the total sample number is 232 cases. The region of interest (ROI) depicting the maximum solid component of the tumor on T2-weighted imaging short time inversion recovery (T2WI-STIR), contrast-enhanced T1-weighted imaging (CE-T1WI), and diffusion-weighted imaging (DWI) images was delineated, and radiomic features were extracted. Feature selection was performed through least absolute shrinkage and selection operator (LASSO) ridge regression, and based on the selected features, six machine learning models including random forest (RF), Extreme Gradient Boosting (XGBoost), support vector machine (SVM), logistic regression (LR), Light Gradient Boosting Machine (LGB) and K-nearest neighbor (KNN) were constructed. The model performance of the training set was verified by using the 5-fold cross-validation method, and the effect evaluation and performance visualization were performed on the test set. After that, the SHAP plot was established based on the feature weights, and finally the benefit degree of patients was analyzed using the DCA curve.

**Results:**

A total of 3375 radiomic features were extracted, and 25 important features were selected after feature extraction to establish six machine learning models. The RF model exhibited the highest performance, achieving an AUC of 0.801, accuracy of 0.800, precision of 0.844, recall of 0.750, and F1 score of 0.794 within the test set. DCA results showed that patients could get good benefits.

**Conclusions:**

The machine learning model based on multimodal MRI radiomic features may serve as a promising tool for predicting the efficacy of chemoradiotherapy in patients with advanced NPC.

## Introduction

Nasopharyngeal carcinoma (NPC) arises from the nasopharyngeal epithelium and ranks as one of the most prevalent malignant tumors affecting the head and neck in clinical settings (1). In comparison to other head and neck tumors, NPC has unique epidemiological, clinical and therapeutic characteristics. According to the relevant data of the International Agency for Research on Cancer, approximately 70% of the new cases of nasopharyngeal cancer worldwide in 2020 occurred in southern China and Southeast Asia (2), with obvious regional clustering. Patients with NPC have complicated symptoms and hidden tumor locations anatomically, and surgery is not commonly the first treatment option for NPC. Currently, the standard treatment approach involves concurrent chemoradiotherapy (CCRT) with or without additional adjuvant chemotherapy (AC) and induction chemotherapy (IC) (3, 4). In particular, with the widespread application of Intensity-modulated radiation therapy (IMRT) in NPC, patients with NPC have entered an era of long-term survival (5). The choice of treatment plan and the prognosis of NPC patients at different stages and among different individuals are also very different. Therefore, evaluating the efficacy of NPC patients after chemoradiotherapy during the treatment course is very important to formulate appropriate treatment plans in real time and thus improve the prognosis of these patients.

Magnetic resonance imaging (MRI), with its high soft tissue resolution, sensitivity and specificity, has been integrated into the entire workflow of nasopharyngeal cancer management, including lesion detection and diagnosis, clinical staging, radiotherapy guidance, and treatment response evaluation (6–8). However, traditional MRI techniques have limited predictive value for the efficacy of chemoradiotherapy in NPC, and the advancement of radiomics, capable of extracting high-dimensional quantitative features from images beyond human visual recognition, presents a novel opportunity for this research area. Radiomics refers to the realization of tumor segmentation, feature extraction and model building by obtaining a large amount of impact information from images; mining, predicting and analyzing image data; and assisting physicians in making the most accurate judgments (9). Therefore, MRI radiomics may provide new insight into the prediction of disease stage, development, curative effect and prognosis. Machine learning, a subset of artificial intelligence, allows computers to process vast datasets using intricate algorithms, recognize data patterns, and continuously enhance models based on training data to produce their predictions (10, 11).

Recently, numerous studies have explored the use of machine learning in clinical practice, making it a standard tool for enhancing the precision of cancer diagnosis and treatment outcomes (12, 13). A series of studies have shown promising outcomes in using machine learning to forecast the survival rate and prognosis of NPC patients (14, 15). However, no relevant studies combining machine learning with MRI radiomics have been found to predict the efficacy of chemoradiotherapy in advanced NPC patients. Therefore, this study aimed to construct a machine learning model based on multimodal MRI radiomics features to predict the efficacy of chemoradiotherapy in advanced NPC patients and detect possible risk factors for local recurrence in a timely manner, which will help clinicians further improve diagnostic and treatment measures and follow-up plans, reduce the recurrence rate of NPC, and improve the prognosis of NPC patients.

## Materials and methods

### Subjects

The clinical and imaging data of 160 patients who were newly diagnosed with advanced NPC via pathology between August 2018 and November 2022, including age, sex, tumor size, bloody nasal discharge, and the presence of lymph node metastasis at initial diagnosis, were retrospectively analyzed.

The inclusion criteria for patients were as follows (1): confirmed pathological diagnosis of nasopharyngeal carcinoma (2); clinical stage II~IV disease (American Joint Committee on Cancer [AJCC] 8th edition) (3); IMRT combined with these chemotherapy methods: CCRT, IC+CCRT, AC+CCRT; and (4) 3.0T magnetic resonance examination 2 weeks before treatment and 2–3 months after treatment.

The exclusion criteria were (1): prior receipt of other antitumor therapies before treatment (2); presence of other primary tumors or severe dysfunction in the heart, liver, kidneys, or other organs; and (3) lack of complete follow-up data.

### Treatment

Every patient received treatment following the nasopharyngeal cancer diagnosis and treatment guidelines of the Chinese Clinical Oncology Association. According to the 2020 Chinese Guidelines for Radiotherapy for Nasopharyngeal Carcinoma, each patient was administered the Intensity-modulated radiation therapy (IMRT) plan. IMRT was performed by 6MV linear accelerator, 2–2.33 Gy each time, 5 times a week, a total of 30–33 times, and the total dose of radiotherapy was 68–76 Gy. Concurrent chemotherapy regimens used a single-drug cisplatin regimen, 100 mg/m²/time cisplatin, intravenous drip, repeated every 21 days and continued from Day 1 to the end of radiotherapy. For patients with advanced NPCa (stage III~IV), simultaneous chemoradiotherapy is combined with induction chemotherapy (IC) or adjuvant chemotherapy (AC). The chemotherapy regimen is based on platinum (cisplatin/nedaplatin). The IC consisted of 2 cycles and was repeated every 21 days. The ACs were repeated every 21 days for a total of 1~3 cycles.

### Evaluation of chemoradiotherapy

MRI of the nasopharynx and neck was performed 2 to 3 months after treatment. The tumor response rate (TRR) was used to evaluate the clinical therapeutic effect of the included NPC patients, and the evaluation criteria were based on the Response Evaluation Criteria in Solid Tumors 1.1 (RECIST 1.1). The tumor regression rate (TRR) = (maximum tumor diameter before treatment - maximum tumor diameter during or after treatment)/maximum tumor diameter before treatment *100%. A TRR=100% corresponded to a complete response (CR), a TRR≥30% indicated a partial response (PR); progressive disease (PD) was documented if the lesion was ≥20% larger than its size before treatment, or if new lesion had emerged, and stable disease (SD) falls fell between partial remission and progression.

### MRI image acquisition

A Philips Achieva 3.0T superconducting magnetic resonance device with dual gradients was employed for serial and functional imaging sequence scans, and image acquisition was carried out with a combined head and neck coil. Position: The supine position was assumed during the scan, with the head first. The patient was asked to breathe naturally during the scan, which extended from the nasopharyngeal skull base to the neck. Parameters: Axis T1WI: TR, 400 ms, TE, 3 ms; axial T2WI: TR, 3500 ms, TE, 83 ms, layer thickness, 2 mm, layer spacing, 0.5 mm, number of excitations (NEX), 1. Axis DWI: SE-EPI sequence, TR, 5100 ms, TE, 70 ms, field of view (FOV), 20 mm×20 mm, layer thickness, 2 mm, layer spacing, 0.5 mm, NEX1, b values, 0 s/mm²and 1000 s/mm². Then, the contrast agent gopenate meglumine at a dose of 0.1 mmol/kg was injected into the cubital vein at a rate of 2 ml/s, and enhanced T2WI-STIR was performed at the axial, coronal and sagittal positions.

### Radiomic features extraction

**Figure 1:**
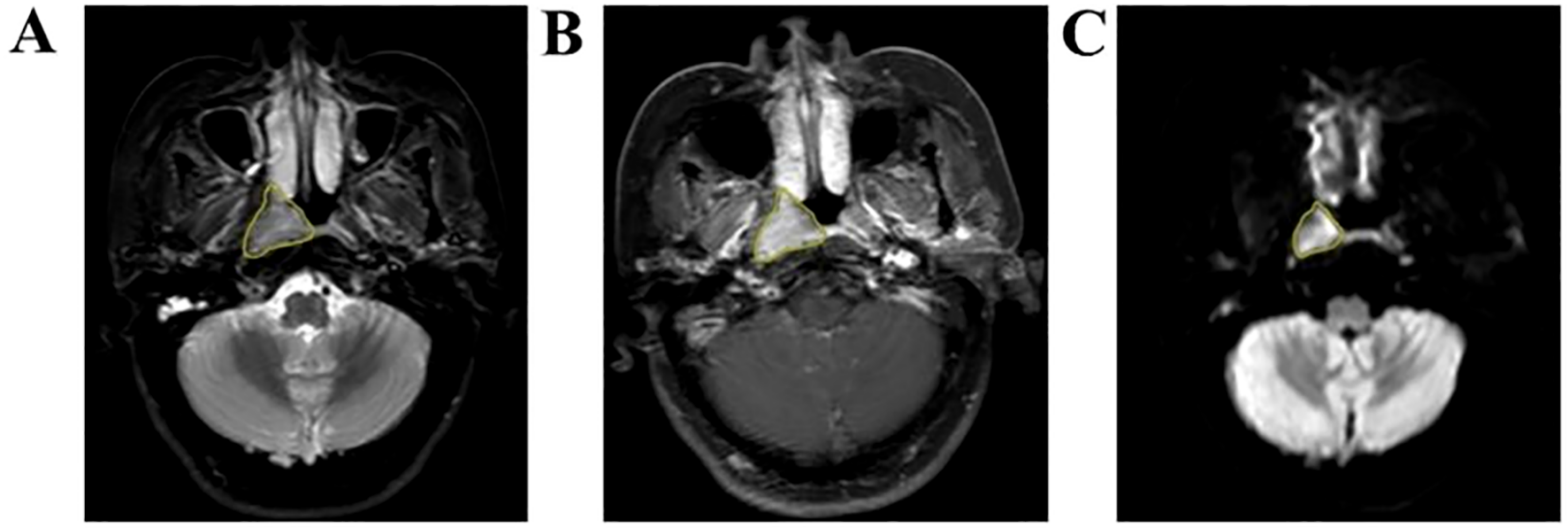
Manual outline of the ROI schematic based on the maximum tumor level. **(A–C)** Results of manual lesion segmentation on T2WI-STIR, CE-T1WI and DWI images, respectively.

Axial T2WI-STIR, CE-T1WI and DWI images of patients 2 weeks before treatment were imported into the medical-Darwin platform in DICOM format. To avoid differences in the sketched target areas, which would result in large differences in radiotherapy effects, all the target areas were read and sketched jointly by two experienced radiology attending physicians and approved by the same chief physician. The method of manual segmentation was selected, and according to the principle of target delineation, the ROI was delineated in the software on the most extensive lesions at the identical level, which exhibited solid tumor components on T2WI-STIR, CE-T1WI, and DWI images (**Figure 1**).

A variety of radiomics features, including shape, first-order, second-order texture, and higher-order based on transformations, were derived from the original multimodal MRI data to generate modified images utilizing the open-source Python library PyRadiomics. A total of 3375 radiomics features were extracted from T2WI-STIR images (n = 1125), CE-T1WI images (n = 1125) and DWI images (n = 1125) for each patient. Second-order texture features encompass the Gray-Level Co-occurrence Matrix (GLCM), Gray-Level Size Zone Matrix (GLSZM), Gray-Level Run Length Matrix (GLRLM), Neighborhood Gray Tone Difference Matrix (NGTDM), and Gray-Level Dependency Matrix (GLDM).

### Radiomics feature selection

**Figure 2:**
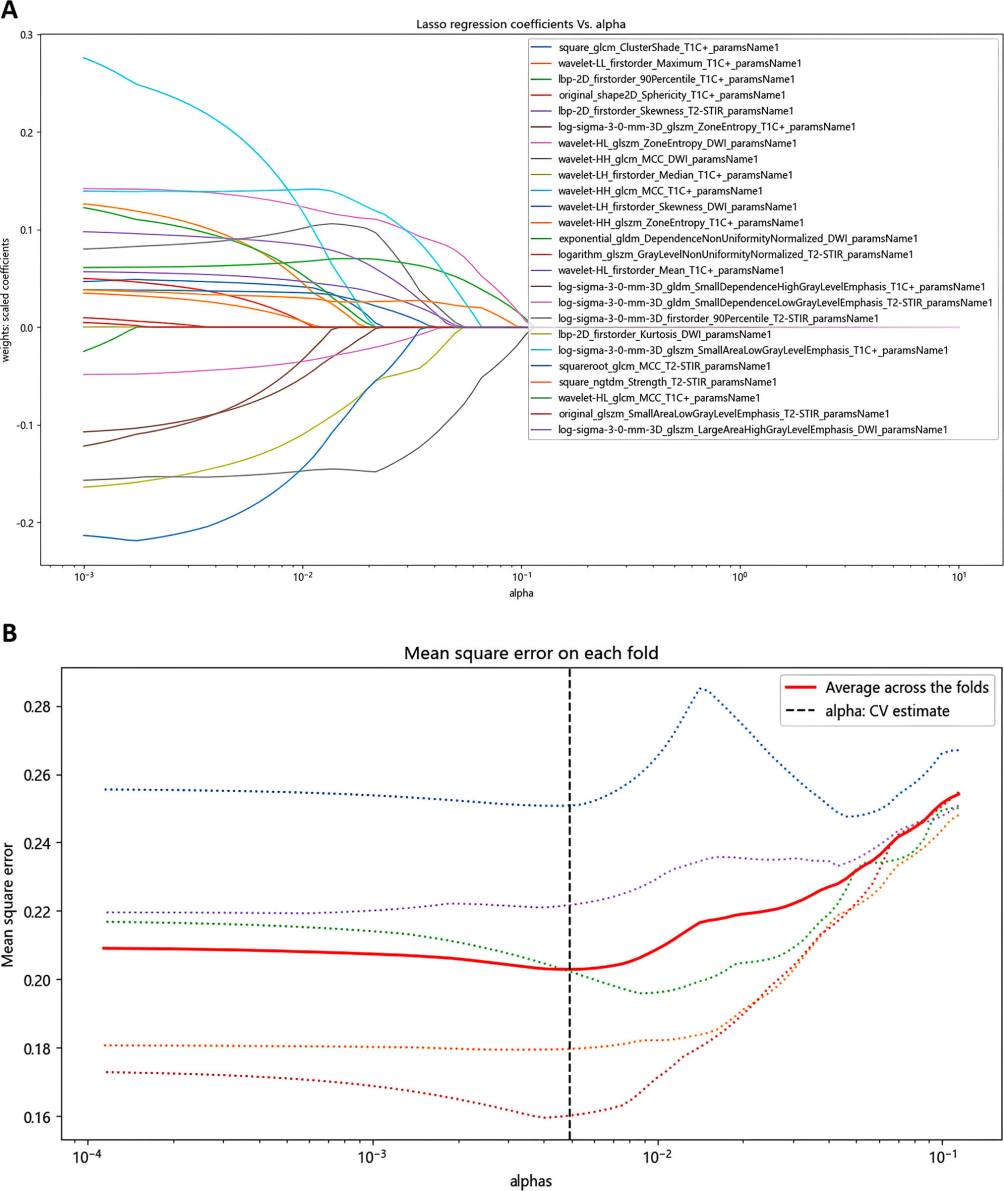
Identification of radiomics features using LASSO screening. **(A)** Curve of regression coefficient with coefficient α. **(B)** LASSO regression mean square change curve for each fold.

The LASSO ridge regression was used for feature selection. Through 5-fold cross-validation, the alpha value with the smallest error is selected as the optimal value of the model, and the feature with non-zero screening coefficient is selected (**Figure 2**). Finally, the 25 most significant features, which hold the greatest predictive value for the effectiveness of chemoradiotherapy in NPC, were selected as the input variables, including 8 T2WI-STIR features, 12 CE-T1WI features and 5 DWI features.

### Machine learning modeling

A total of 160 patients in the dataset were divided into an effective group (n=116) and an ineffective group (n=44). After applying SMOTE for sample balancing, the effective and non-effective groups were adjusted to a 1:1 ratio (116 cases each), resulting in a total sample size of 232 cases. The dataset is divided into a training set (n=162) and a test set (n=70) in a 7:3 ratio. Based on the features selected, six machine learning models, including Random Forest (RF), Extreme Gradient Boosting (XGBoost), Support Vector Machines (SVM), Logistic Regression (LR), Light Gradient Boosting Machine (LGB), and K-Nearest Neighbor (KNN), are built. The performance of the models is verified using a 5-fold cross-validation method, and the effectiveness and performance of the models on the test set are evaluated and visualized. The model’s clinical net benefit was assessed using decision curve analysis (DCA).

### Statistical analysis

The data were statistically analyzed using SPSS Statistics version 26.0. The Kolmogorov-Smirnov test was applied to determine the data’s distribution normality. Measurement data that follow a normal distribution are expressed as the mean ± standard deviation, with independent samples t-tests used for comparisons between groups. For data that do not conform to a normal distribution, they are depicted as [M50(P25, P75)]. The count data are expressed as [n (%)]. P<0.05 was considered statistically significant.

## Results

### Clinical characteristics of the patients

After statistical analysis (**Table 1**), statistically significant differences were found between the effective group and the noneffective group in terms of the presence or absence of bloody nasal discharge, T stage and clinical stage (P < 0.05), while no statistically significant differences in age, sex, tumor size, or lymph node metastasis at initial diagnosis were identified (P > 0.05).

**Table 1:** Demographic characteristics of the study population.

Characteristics	Total (n=160)	Effective group (n=116)	Noneffective group (n=44)	*P*
Age (year)	52.0 ± 12.8	51.6 ± 12.8	52.9 ± 12.6	0.581
Sex, n (%)				0.769
Female	41 (25.6)	29 (25.0)	12 (27.3)	
Male	119 (74.4)	87 (75.0)	32 (72.7)	
Tumor size (cm)	3.2 [2.7, 3.6]	3.2 [2.7, 3.6]	3.1 [2.6, 3.6]	0.439
Bloody nasal discharge, n (%)				0.043
No	96 (60.0)	64 (55.2)	32 (72.7)	
Yes	64 (40.0)	52 (44.8)	12 (27.3)	
Lymph node metastasis, n (%)				0.594
No	14 (8.8)	11 (9.5)	3 (6.8)	
Yes	146 (91.2)	105 (90.5)	41 (93.2)	
T stage, n (%)				0.016
T1-T2	83 (51.9)	67 (57.8)	16 (36.4)	
T3-T4	77 (48.1)	49 (42.2)	28 (63.6)	
Clinical stage, n (%)				0.010
II~III	121 (75.6)	94(81.0)	27 (61.4)	
IV	39 (24.4)	22(19.0)	17 (38.6)	

The data are presented as the mean ± S.D. or number (%).

### Feature selection

A cumulative total of 3375 radiomics features were derived from the multimodal MRI scans for each patient, with 1125 features obtained from T2WI-STIR, 1125 from CE-T1WI, and 1125 from DWI sequences. We used LASSO Ridge regression for feature selection, and extracted 25 important features from 3375 radiomic features as input features, including 2 shape features, 8 first-order features, 3 GLDM features, 5 GLCM features and 7 GLSZM features.

### Prediction performance of models

**Figure 3:**
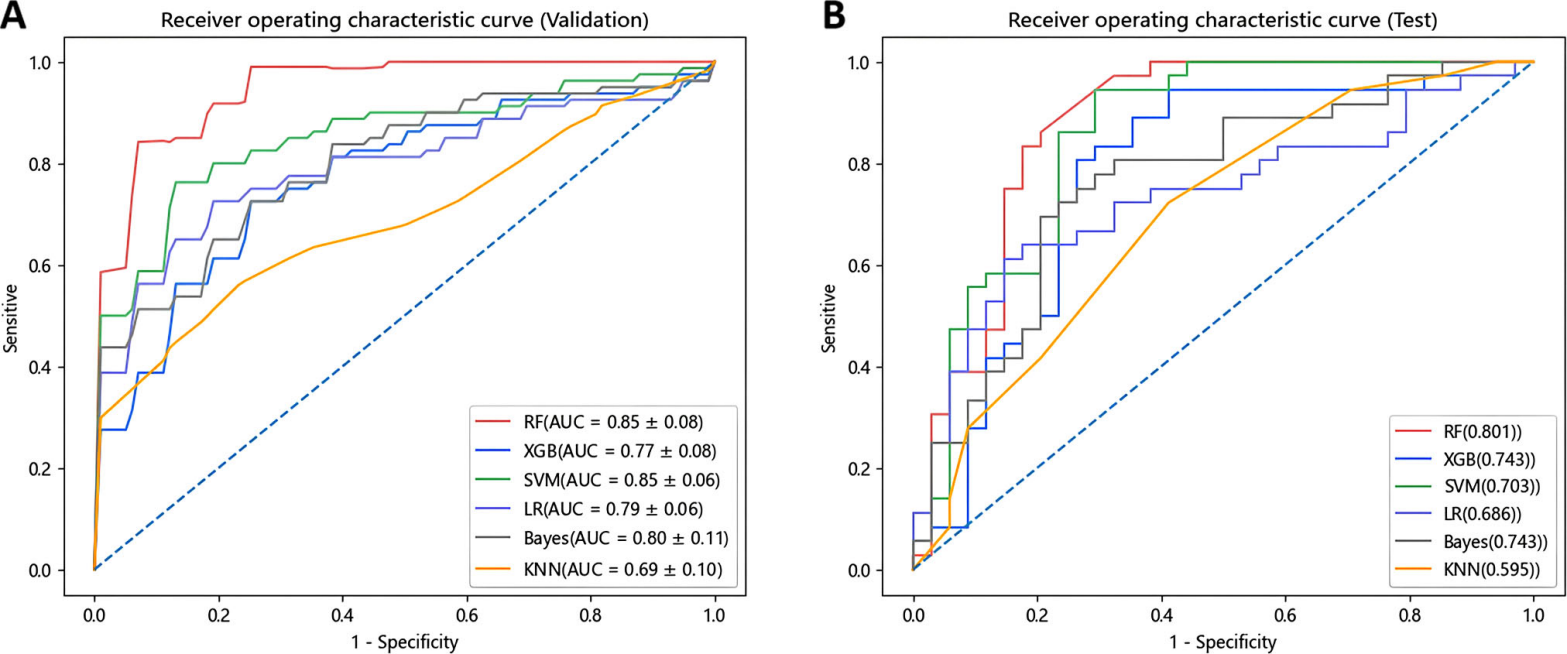
ROC curves of six machine learning models on the validation set **(A)**, and test set **(B)**.

**Figure 4:**
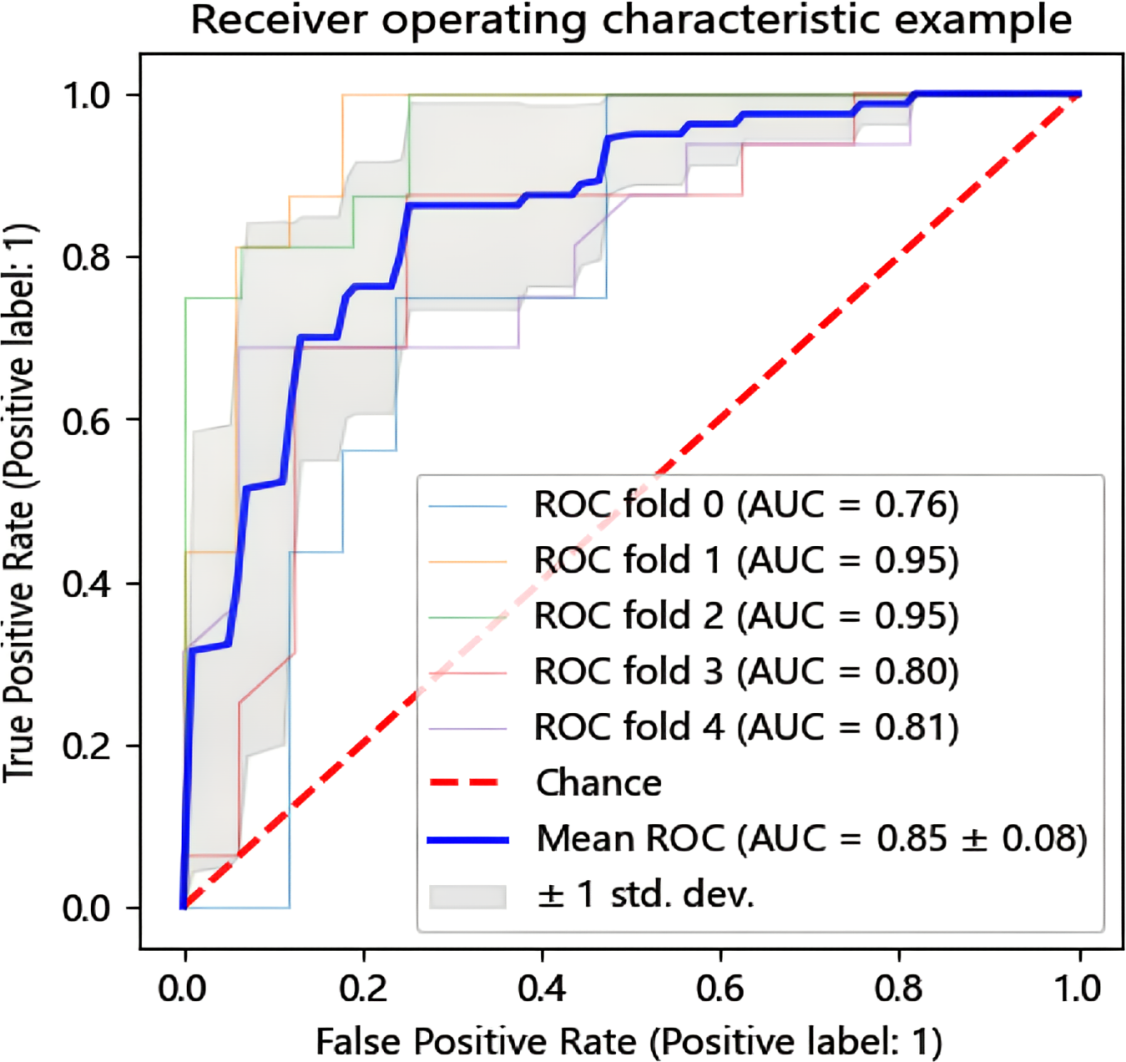
The ROC curve is validated by the random forest model with a 5-fold cross.

In this research, we employed six distinct machine learning algorithms to construct a radiomic predictive model within the training set and subsequently validated its reliability. ROC curves of various radiomic models predicting the efficacy of chemoradiotherapy for NPC in the validation set and the test set are shown in **Figure 3**. The Random Forest (RF) model had the best performance, with AUC of 0.801, accuracy of 0.800, precision of 0.844, recall rate of 0.750 and F1 score of 0.794 in the test set (**Table 2**). The model performance was verified by using the 5-fold cross-validation method (**Figure 4**). Then, we used the Delong test to compare the differences in AUC between each model and the RF model (**Table 3**).

**Table 2:** Six machine learning models test set performance.

Model	Accuracy	Recall	Precision	F1	AUC
RF	0.800	0.750	0.844	0.794	0.801(0.698-0.905)
XGB	0.743	0.722	0.765	0.743	0.743(0.628-0.859)
SVM	0.700	0.583	0.778	0.667	0.703(0.582-0.825)
LR	0.686	0.667	0.706	0.686	0.687(0.562-0.810)
LGB	0.743	0.722	0.765	0.743	0.743(0.628-0.859)
KNN	0.586	0.278	0.769	0.408	0.598(0.462-0.728)

**Table 3:** Delong test between models.

comparison of models	Log10 (P value)	P value
XGB-RF	-0.5657	0.2726
SVM-RF	-1.1803	0.0661
LR-RF	-1.0891	0.0815
LGB-RF	-0.6282	0.2358
KNN-RF	-3.697	0.0002

### Establishment of DCA curve

**Figure 5:**
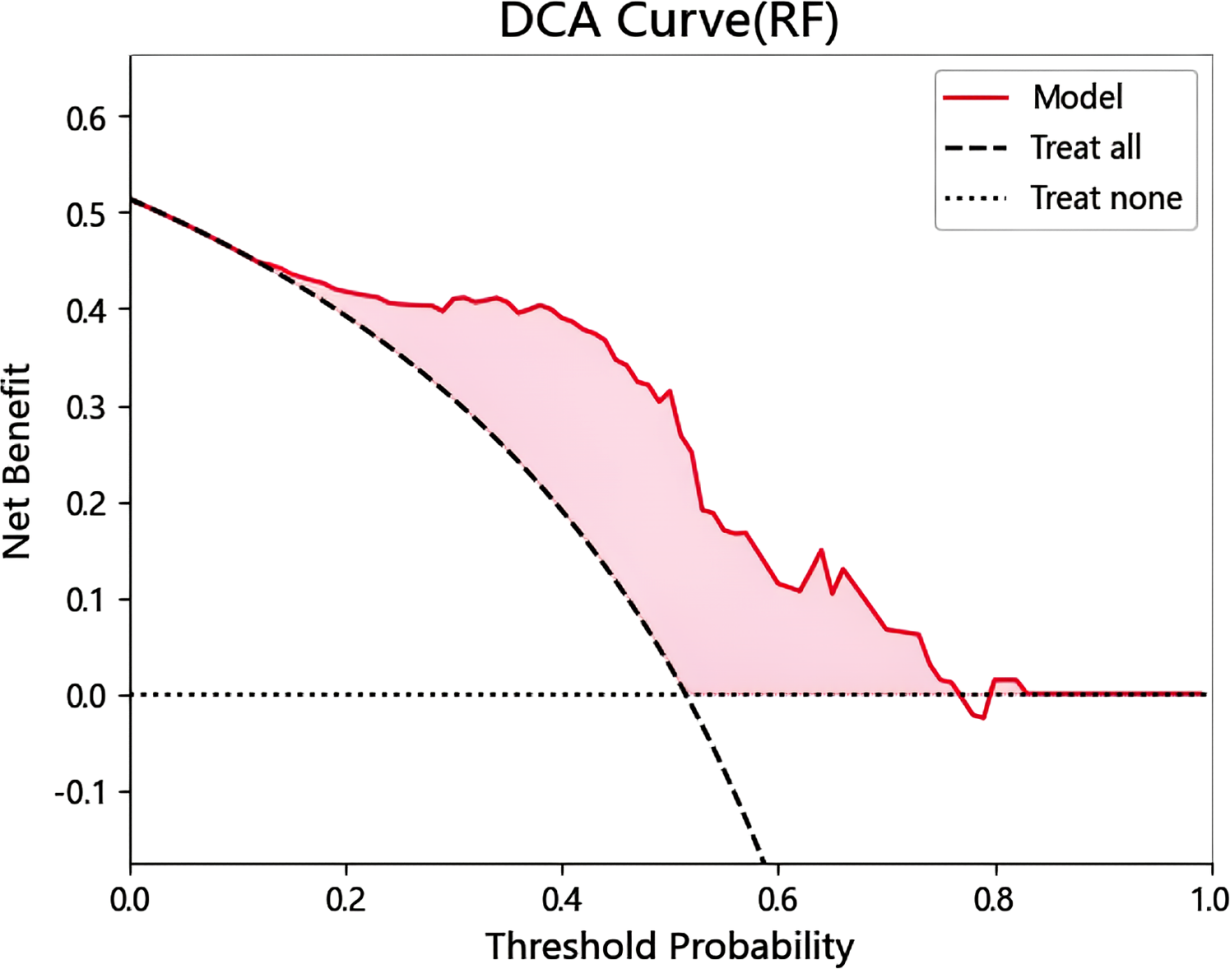
Decision curve of RF model test set.

DCA analysis was performed to evaluate prognostic decision, and the application of RF classifier model had good clinical applicability (**Figure 5**). It can be known from the DCA curve that when the threshold probability is within the range of 0.17 to 0.77, the model decision curve is above the extreme curve, and the corresponding value of the net benefit on the vertical coordinate is 0 to 0.44. That is, when the model predicts a disease probability of 17% to 88%, among 100 patients, 0% to 44% of them can benefit after the intervention of doctors.

### Establishment of SHAP plot

**Figure 6:**
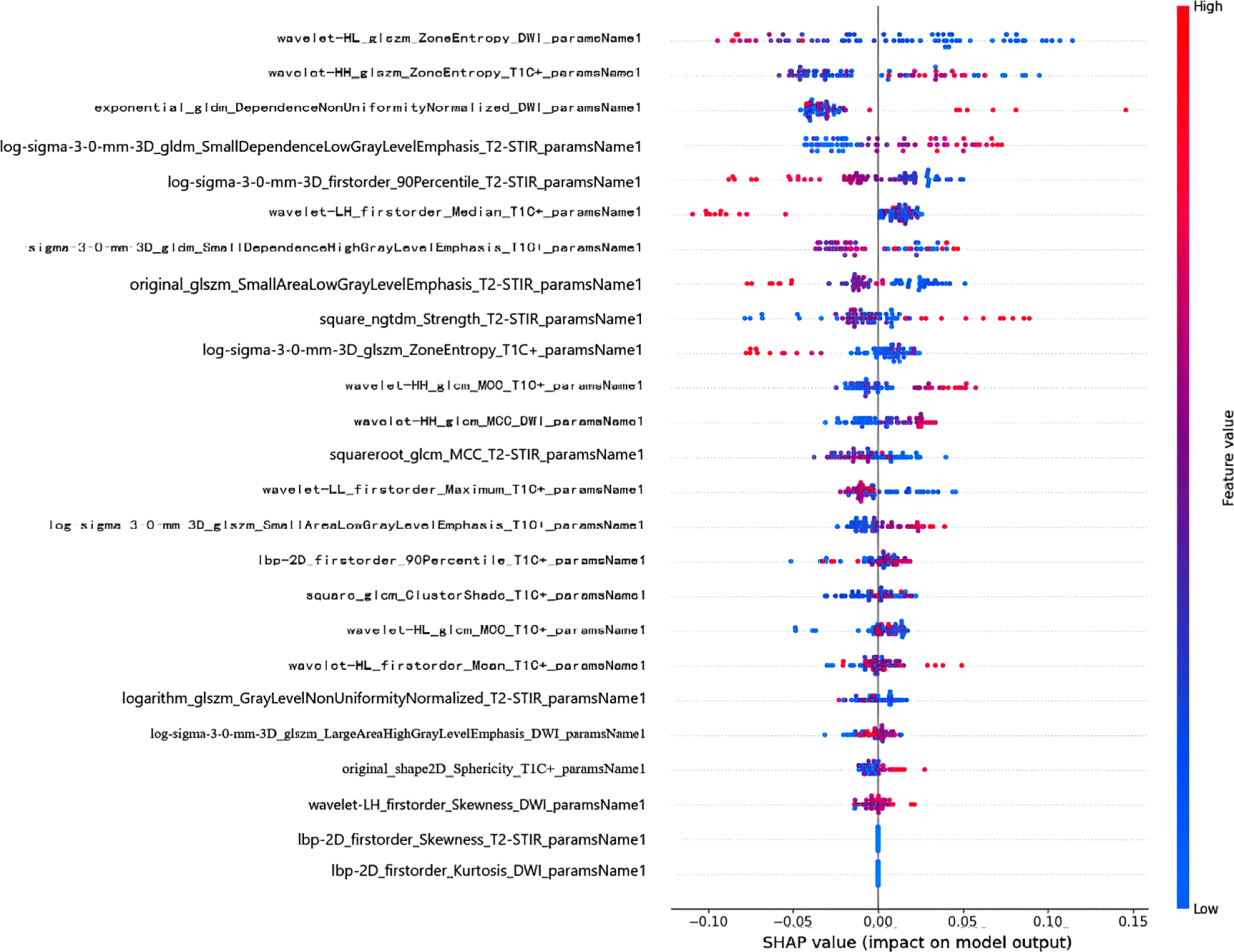
SHAP summary plot of RF model. The x-axis represents the SHAP values, reflecting the impact of each feature on the model’s predictions.

In order to reveal the internal decision mechanism of the model, SHAP method is used for interpretability analysis. By comparing the SHAP values of various features, the contribution of different features to the RF model impact can be intuitively seen. Each data point is colored from low (blue) to high (red) according to the value of the feature. The further to the right the point is, the greater the positive impact of the feature on the model output. The further to the left the dot is, the greater the negative impact will be. Through the Shapley graph in this study, it is found that the wavelet transform features extracted based on DWI sequence have the highest Shap distribution interval. As illustrated in **Figure 6**, wavelet-HL_glszm_ZoneEntropy_DWI_paramsName1 was the top feature that contributed the most to the model.

## Discussion

At present, the treatment plan for NPC is moving toward individualized and precise treatment (16); that is, according to the varying sensitivity of different patients to treatment, individualized treatment plans are formulated to accurately evaluate the efficacy of chemoradiotherapy, which plays a crucial role in guiding the comprehensive treatment and prognosis of NPC. Given the need for individualized precision treatment for NPC, we developed a machine learning model based on MRI radiomics to predict the efficacy of chemoradiotherapy for NPC. The results of this study demonstrate the potential of the RF model, which achieved good recognition performance with an AUC of 0.801, accuracy of 0.800, precision of 0.844, recall rate of 0.750 and F1 score of 0.794 on the test set. Guiding the comprehensive treatment of advanced NPC patients is important to avoid inadequate treatment or overtreatment. The highlights of this study are as follows (1): only MRI radiomics features were used for modeling (2); feature extraction was performed using T2WI-STIR, CE-T1WI and DWI images; and (3) the machine learning model was used to conduct 5-fold cross-validation, and the results were more reliable.

Predictive models based on radiomic features have been widely used to evaluate therapeutic effects on various diseases, providing a noninvasive method for evaluating patient prognosis (17–19). In previous studies, pretreatment MRI features were used to construct a model to predict the efficacy of chemoradiotherapy for NPC to enable treatment plan modification at an early stage (20–22). Zhao et al. (22) combined clinical data with radiomic features generated by support vector machines to build a radiomic nomogram. The results showed that radiomic nomogram based on MRI-based multi-parameter imaging is helpful for individualized risk stratification and treatment of NPC patients receiving IC. However, most of these studies used conventional T1WI and T2WI for radiomics feature analysis and did not include DWI images for sketching; therefore, the image information was incomplete. DWI, as a routine part of nasopharyngeal MRI protocols, could play a crucial role in assessing the treatment response and prognosticating outcomes for NPC (23). Within this research, 25 optimal radiomics features were extracted based on multimodal MR images. Out of the 25 features used for model construction, 8 were derived from T2WI-STIR, 12 from CE-T1WI, and 5 from DWI were selected, which preliminarily indicated that DWI parameters are of comparable importance. In addition, through the Shapley graph in this study, it is found that the features extracted based on DWI sequence have the highest Shap distribution interval. Studies (24) have shown that CE-T1WI can reflect the tumor microenvironment and tumor aggressiveness by displaying microvascular density and perfusion, and T2WI can provide tumor morphology and interstitial information. DWI offers enhanced subvoxel-level details on tumor heterogeneity, capturing the restricted Brownian motion and tumor microstructure (25, 26). Compared with other radiomics studies, this experiment extracted more morphological, textural and transformed high-throughput radiomic features from the included patient multimodal images and obtained more abundant image features to maximize the detailed features of the original images.

Previous studies often used multiple data sources (such as demographic data, hematological tests, and vital signs) to develop models predicting treatment efficacy and prognosis for NPC (27–29). However, similar to findings in most other studies that have established predictive models based on clinical data, patients with NPC may also be diagnosed with a number of other diseases that may cause changes in the clinical markers being studied. In this study, we used only MRI radiomics features for modeling, which simplified the modeling process and increased the reliability of the data and research results. In addition, traditional studies predicting adverse outcomes or treatment responses are mostly based on multivariate logistic regression models (30–32), which limit the number of features that can be used due to the linear assumption between predictors and outcomes. In contrast, machine learning-based models can use more parameters when addressing complex relationships between predictors and outcomes (10). In this study, we selected 6 machine learning models commonly used in medical problems, Among them, the test set accuracy, precision and AUC values using RF model are the highest, which is the optimal model in this study. RF model introduces randomness, increases the diversity of the model, and can effectively process high-dimensional data without being affected by collinearity, which lays a foundation for the clinical significance of this study in data analysis, and makes it have certain clinical application value. In addition, in traditional research, data sets are usually fixed, divided into training sets and validation sets, most of which do not carry out cross-validation (33, 34). In this study, the total sample of 160 cases after the overall SMOTE sample balance, positive and negative sample ratio of 1:1, 116 cases, the total sample number of 232 cases. Then, the data set was divided into training set and test set according to the ratio of 7:3, and 5-fold cross-validation was adopted during training to avoid contingencies in model evaluation. We analyzed and integrated the data through a combination of radiomics and machine learning, and the trained model is more reliable than traditional logistic regression models.

The study also had some limitations. First of all, due to limitations in case collection, this study did not use an external test set to evaluate the model. Future research should focus on multicenter studies to further validate model performance and increase model generalizability. Second, this study only included patients with stage II-IV NPC, and patients with early stage NPC should be included in the future. Third, Only the radiomic features of a single sequence of MRI before treatment were extracted, and multi-parameter and multi-time sequence MRI analysis could not be carried out. In future studies, we can try to leverage the comprehensive data of multi-parameter and multi-time sequence MRI. Fourth, This research did not delve into the specific impacts of various chemotherapy protocols on patient outcomes, and future studies should conduct more detailed subgroup analyses on distinct chemotherapy regimens and drugs. Finally, in this research, only the two-dimensional ROI at the most extensive level was delineated, which may have led to the loss of some tumor information. Adding some 3D features may increase the diagnostic efficiency of the model, which is worthy of further study.

## Conclusion

In conclusion, we have established and verified a predictive model for chemoradiotherapy outcomes in patients with advanced NPC, utilizing multimodal MRI radiomic features coupled with machine learning algorithms. Should our findings be substantiated in future multi-center studies, this approach could emerge as a non-invasive predictive instrument to assess the response to chemoradiotherapy in advanced NPC patients. This would furnish clinicians with crucial data to formulate more effective treatment strategies, thereby enhancing clinical decision-making in the realm of personalized precision medicine.

## Data Availability

The original contributions presented in the study are included in the article/supplementary material. Further inquiries can be directed to the corresponding author.
